# Establishment of a novel mouse xenograft model of human uterine leiomyoma

**DOI:** 10.1038/s41598-018-27138-1

**Published:** 2018-06-11

**Authors:** Yusuke Suzuki, Masaaki Ii, Takashi Saito, Yoshito Terai, Yasuhiko Tabata, Masahide Ohmichi, Michio Asahi

**Affiliations:** 1 Department of Obstetrics and Gynecology, Faculty of Medicine, Osaka Medical College, Osaka, Japan; 2 Division of Research Animal Laboratory and Translational Medicine, Research and Development Center, Osaka Medical College, Osaka, Japan; 3 Department of Pharmacology, Faculty of Medicine, Osaka Medical College, Osaka, Japan; 4 Department of Legal Medicine, Faculty of Medicine, Osaka Medical College, Osaka, Japan; 5 Laboratory of Biomaterials, Department of Regeneration Science and Engineering, Institute for Frontier Life and Medical Sciences, Kyoto University, Kyoto, Japan

## Abstract

Uterine leiomyoma is the most common benign tumour in women, and an appropriate animal model for leiomyoma would be useful for exploring new therapeutic strategies. Therefore, we have been challenged to develop a new simple mouse model for human leiomyoma. Leiomyoma tissues were harvested from myomas resected by different surgical procedures with or without gonadotropin-releasing hormone agonist (GnRHa) treatment and were subcutaneously implanted into BALB/c nude mice with an estradiol/progesterone-releasing pellet. The implanted leiomyoma tissues that were obtained from the marginal site of large myomas resected by abdominal myomectomy with GnRHa treatment exhibited sufficient tumour growth in the transplanted mice. The leiomyomas that were treated with GnRHa highly expressed the estrogen/progesterone receptor genes, insulin-like growth factor 2 (IGF2) and embryonic smooth muscle myosin heavy chain (SMemb), which suggests that these factors are critical in the establishment of a mouse model of growing leiomyoma. As a result, this model will be useful for the development of new therapeutic strategies.

## Introduction

Uterine leiomyomas (also called fibroids or myomas) are the most common gynaecologic tumours in women and are a major reason for hysterectomy in Western countries^1,2^. It is also known that ethnic differences exist in the incidence of uterine leiomyomas. In African-American women, the incidence of uterine myomas is 60% by age 35 and increases to over 80% by age 50. In contrast, Caucasian women have an incidence of 40% by age 35 and almost 70% by age 50^3^. Little direct evidence has been published, but Asian women appear to show an intermediate incidence rate between that of African-Americans and Caucasians; this observation is based on the genetic profiling of leiomyomas^4^. Although uterine myomas are benign tumours and most of them cause no symptoms, many women experience significant clinical symptoms such as abnormal uterine bleeding, pelvic pressure/pain, and reproductive dysfunction, which warrant certain therapies.

With respect to current therapies for leiomyomas, the treatment options vary and are typically individualized based on the severity of the symptoms, the size and location of the leiomyoma lesions, the patient’s age, chronological proximity to menopause, and the patient’s desire for future fertility^5^. The standard treatment for leiomyoma is surgical intervention, and although hysterectomy is the definitive surgical therapy, myomectomy is still commonly performed, especially in women who desire future fertility. More recently, developed techniques, which include uterine artery embolism (UAE)^6^, magnetic resonance-guided focused-ultrasound surgery (MRgFUS)^7^, and myolysis^8^, are emerging as minimally invasive alternative surgical therapies. On the contrary, medical treatment using hormone-based agents (e.g., oral contraceptives, levonorgestrel-containing intrauterine systems (IUSs) and gonadotropin-releasing hormone (GnRH) agonists are also available. While these therapies provide varying degrees of control for abnormal uterine bleeding, most of them do not act directly on the leiomyomas, and no definitive agents for the long-term medical treatment of uterine leiomyoma have been developed. In the recent past, the off-label use of GnRH agonists with or without hormonal therapy has been the standard of care. Although other agents are being investigated in clinical trials, few promising therapeutic agents have been developed for the treatment of uterine leiomyomas^9^. For this reason, the development of an inexpensive agent with the ability to shrink leiomyomas with minimal to no side effects and that does not interfere with ovulatory cycles or fertility potential is required.

One of the major reasons for the unmet progress in the development of novel, promising therapeutic agents and next generation novel strategies is the lack of an appropriate animal model of human leiomyoma. Most of the recently developed mouse xenograft models for human leiomyoma require severe immunodeficient mice and growth factor-containing gels with a piece of human leiomyoma tissue^10^ or certain cell lines with genetic modifications^11–13^. In this study, we were challenged to develop a more simplified/appropriate mouse model for growing human leiomyoma. We focused on the size, pretreatment with or without GnRHa, and the portions (central/marginal) of the leiomyoma that were excised.

## Results

### Matrigel^TM^ reduced the size of transplanted leiomyomas with increased vascularity

**Figure 1:**
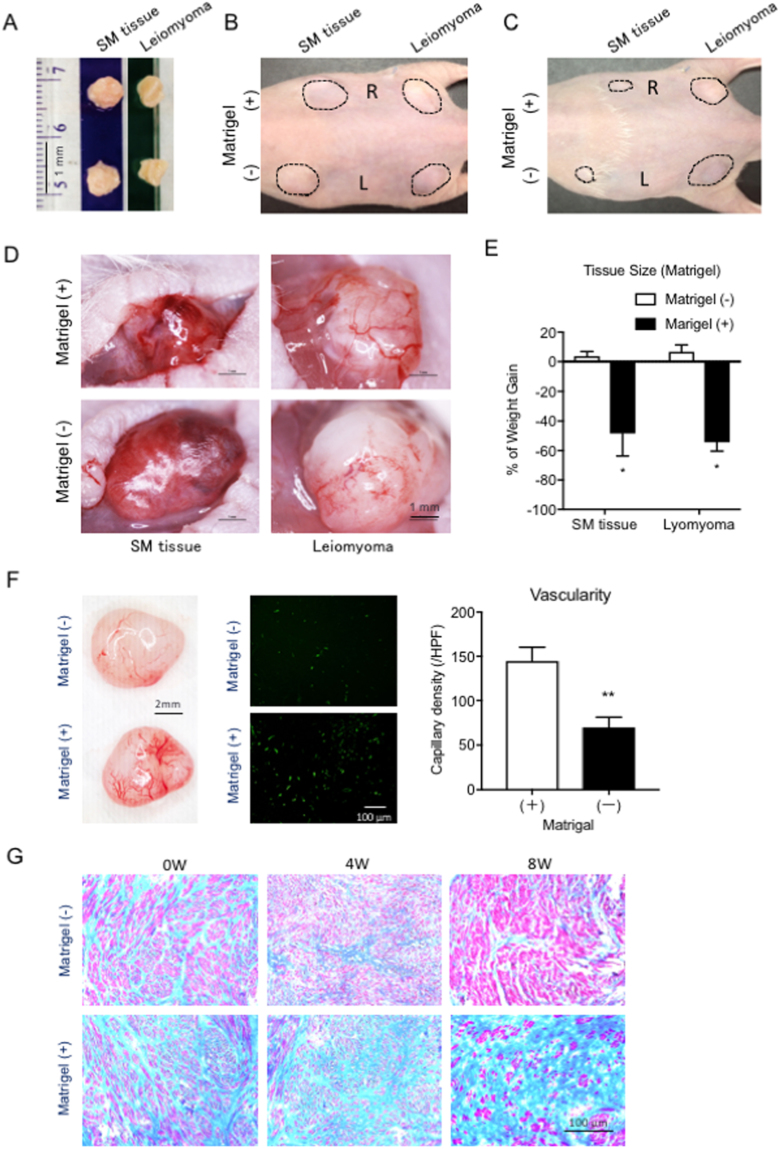
Transplantation of tissue pieces isolated from normal human uterine wall and leiomyoma with or without Matrigel^TM^. (**A**) Two tissue pieces (5 mm in diameter) were resected from normal uterine smooth muscle (SM) tissue (left) and leiomyoma (right) in the same hysterectomized uterus. (**B**) The pieces of SM tissue and leiomyoma were transplanted with (R, right side) or without Matrigel^TM^ (L, left side) under the dorsal skin of a mouse. (left panel) The dotted line-circles indicate the transplanted sites of the tissue pieces. (**C**) The transplanted pieces of SM tissue and leiomyoma with or without Matrigel^TM^ under the dorsal skin of a mouse were macroscopically observed 4 weeks after transplantation. (**D**) The transplanted tissue pieces in the dorsal skin (**C**) were exposed following a skin incision made with scissors. (**E**) The percent of weight gain in the mice with transplanted SM tissue and leiomyoma with or without Matrigel^TM^ at 4 weeks after transplantation. (*p < 0.05 vs. Matrigel^TM^ (−), n = 3). (**F**) The resected transplanted pieces of leiomyoma with or without Matrigel^TM^ (left panel) and immunostaining images of the tissue sections with isolectin B4 (ILB4, green) (center panels). The vascularity was expressed as capillary (ILB4-positive dot) density counted at high power magnification. (right panel) HPF, high power field. (**p < 0.01 vs. Matrigel^TM^ (−), n = 5) (**G**) Masson’s Trichrome- stained tissue sections from leiomyoma with (+) or without (−) Matrigel^TM^ before (0 W), 4 and 8 weeks after transplantation. Pink, normal tissue and blue, fibrotic tissue.

We first simply transplanted a normal uterine muscle layer and leiomyoma tissue (Fig. 1A) with or without Matrigel^TM^ in a BALB/c nude mouse (Fig. 1B) in order to see if and how Matrigel^TM^ influences the transplanted tissue. As expected, increased vascularity was observed in both the transplanted uterine muscle layer and leiomyoma, but unexpectedly, the size of the transplanted tissues was reduced both in the uterine muscle layer and in the leiomyoma when Matrigel^TM^ was added. (Fig. 1C–E) On the contrary, both the size of the transplanted uterine muscle layer and that of the leiomyoma were maintained at their original size with reduced vascularity without Matrigel^TM^ compared with those with Matrigel^TM^. (Fig. 1C–E) We then further examined vascularity in the leiomyoma tissue histologically. The capillary density was significantly reduced in the xenograft transplanted with Matrigel^TM^. (Fig. 1F) In addition, the extent of fibrosis in the leiomyoma tissue when Matrigel^TM^ was used was increased, while that without Matrigel^TM^ was decreased at 4 and 8 weeks following transplantation. (Fig. 1G) Based on these observations, we decided not to use Matrigel^TM^ at the time of transplantation of leiomyoma tissue in the following experiments.

### Supplementation of estradiol and progesterone successfully increased the size of transplanted leiomyomas

**Figure 2:**
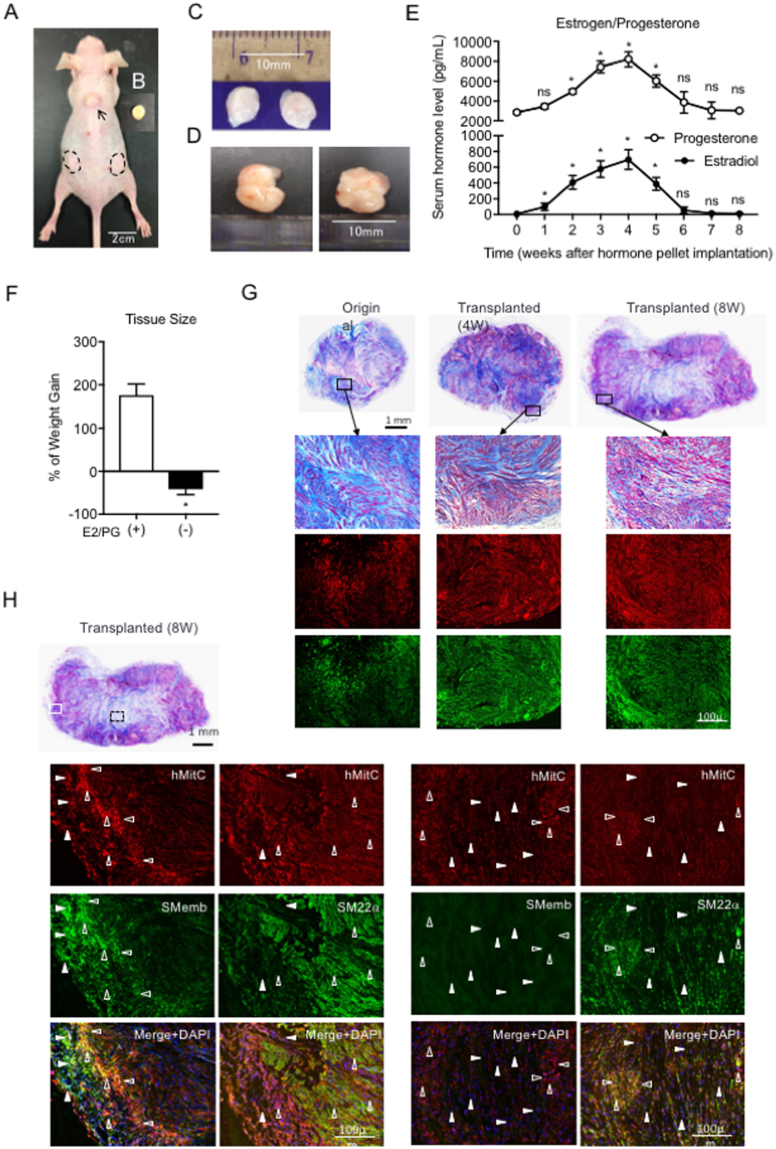
Establishment of the transplantation of human leiomyoma and estradiol/progesterone pellet in BALB/c nude mice. (**A**) A BALB/c nude mouse with 2 pieces of human leiomyoma (dotted line-circle) and a 17beta-estradiol/progesterone pellet (arrow) 6 weeks after transplantation. (**B**) The pellet contains 1.5 mg of 17beta-estradiol and 5 mg of progesterone within a PLGA biodegradable matrix. (**C**) The initial pieces of human leiomyoma resected from a hysterectomized uterus (50 mg/each). (**D**) The resected transplanted leiomyoma tissues (left, 176 mg and right, 151 mg) 6 weeks after transplantation. (**E**) The time course of serum estradiol and progesterone levels in mice implanted with an E2/PG-PLGA pellet. (ns and *P < 0.05 vs. 0 weeks). (**F**) The percent of weight gain in mice with the transplanted leiomyoma tissue (n = 3) with (E2/PG (+)) or without (E2/PG (−)) the E2/PG-PLGA pellet at 8 weeks after tissue transplantation. (*p < 0.05 vs. E2/PG (+). (**G**,**H**) Masson’s trichrome-stained tissue sections from entire and high-magnification images of transplanted leiomyoma before (Original), 4 weeks (Transplanted (4 W)) and 8 weeks after transplantation (Transplanted (8 W)). (**G**) Double-immunofluorescent staining for Caldesmon (red) and human mitochondrial ribosomal protein L11 (hMitC, green) in the original leiomyoma, xenograft tissue sections at 4 weeks (Transplanted (4 W)), and those at 8 weeks (Transplanted (8 W)) following transplantation. (**H**) Double-immunofluorescent staining for hMitC (red) and SMemb (green) or SM22α (green). Closed arrowheads indicate infiltrated cells originated from mouse (host side: hMitC-negative) and open arrowheads indicate grown cells originated from human leiomyoma tissue (donor side: hMitC-positive). In Masson’s trichrome-stained tissue sections from entire transplanted leiomyoma 8 weeks after transplantation, the lesion with white rectangle corresponds to the left 6 panels and black rectangle corresponds to the right 6 panels.

Since recent accumulating evidence has revealed that both estradiol and progesterone are required for the development of leiomyoma, we followed a protocol that included the use of both estradiol and progesterone. Dr. Tabata (Kyoto University, Japan) generated the 17beta-estradiol- and progesterone-containing PLGA pellet (Fig. 2B), which was biodegradable and used for controlled drug release *in situ* in our experiments. The two pieces of leiomyoma tissue (Fig. 2C), which were transplanted along with a PLGA pellet that incorporated 17beta-estradiol and progesterone, were placed under the dorsal skin in BALB/c nude mice. (Fig. 2A, at 4 weeks after transplantation) After 4 and 8 weeks, the transplanted tissue pieces (Fig. 2D) were harvested and examined histologically. The serum levels of estradiol and progesterone gradually increased to approximately 800 pg/mL and 8000 pg/mL, respectively, at 6 weeks following implantation of the E2-PLGA and PG-PLGA pellets; these levels peaked at 4 weeks after pellet implantation. (Fig. 2E) The weight gain was evaluated in the mice with transplanted leiomyoma tissues with or without the E2/PG-PLGA pellet at 8 weeks following pellet/tissue implantation, and it was observed that E2/PG supplementation successfully led to an increase in weight. (Fig. 2F) Although the leiomyoma tissue sections (Masson’s trichrome staining) from the original and the transplanted tissue (4 and 8 weeks after transplantation) exhibited similar histological findings, the fibroblasts stained in blue were distributed evenly at 4 weeks and mainly localized in central site at 8 weeks after making xenograft. To confirm the cellular origins of the xenograft, we performed double fluorescent immunostaining for human specific mitochondrial ribosomal protein (hMitC) and SMC specific caldesmon. The hMitC (green)- and caldesmon (red)-positive cells were also distributed evenly at 4 weeks, whereas those were localized in marginal site at 8 weeks after making xenograft. These findings were also compared to those of Masson’s trichrome staining images. (Fig. 2G) In addition, we performed another double immunofluorescent staining for hMitC and SMemb, a marker for synthetic/dedifferentiated SMC/myofibroblast or SM22α. It was revealed that the xenograft consisted of both infiltrated cells originated from mouse (host) and grown cells originated from human leiomyoma tissue (donor). Interestingly, SMemb-positive cells co-localized with hMitC-positive cells were mainly distributed in marginal site of xenograft, while SM22α-positive SMCs were mainly distributed in relatively inner side of xenograft at 8 weeks after implantation, suggesting that transplanted leiomyoma was growing toward outside with synthetic/dedifferentiated SMCs or myofibroblasts. (Fig. 2H, left panels) In contrast, less number of hMitC/SM22α-double positive cells and no SMemb-positive cells could be observed in central site of xenograft. (Fig. 2H, right panels) These findings indicate that the transplanted human leiomyoma tissue developed *in situ* changing its original histological identity with infiltrated mouse-derived cells.

### Surgical procedure, GnRHa treatment, and size of the leiomyoma influenced the growth of transplanted leiomyomas *in vivo*

**Figure 3:**
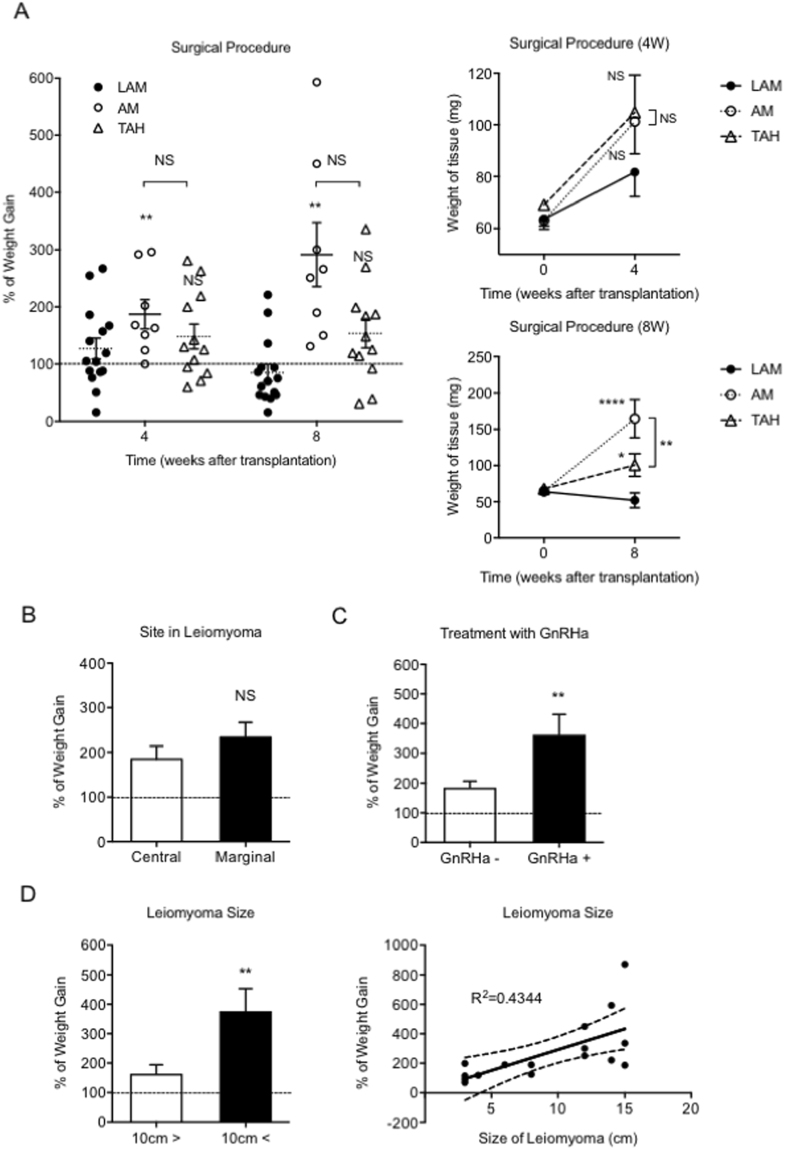
Differential growth rate of the transplanted human leiomyoma tissue among three different surgical procedures. (**A**) The leiomyoma tissues were harvested from samples obtained by laparoscopically assisted myomectomy (LAM, n = 15), abdominal myomectomy (AM, n = 8), and total abdominal hysterectomy (TAH, n = 16) and were transplanted in BALB/c nude mice (n = 39) according to an established procedure. The leiomyoma tissue growth rate was expressed as the percent of weight gain in a scatter plot (left panel); also shown are the weight of the tissue (mg) 4 weeks (right upper panel) and 8 weeks (right lower panel) following transplantation. (NS, not significant and *P < 0.05; **P < 0.01; ****P < 0.0001 vs. LAM). (**B**) The leiomyoma tissues were collected from the central site (Central, n = 10) and the marginal site (Marginal, n = 10) of myomas and transplanted according to the established surgical procedure; the average percent weight gain was assessed. (NS, not significant vs. Central). (**C**) The leiomyoma tissues were collected from the marginal site of myomas resected from patients with (GnRHa + , n = 10) or without GnRHa treatment (GnRHa−, n = 10) and were transplanted according to the established surgical procedure; the average percent weight gain was assessed. (**P < 0.01 vs. GnRHa−). (**D**) The leiomyoma tissues were collected from small (<10 cm, n = 10) and large (>10 cm, n = 9) myomas and were transplanted according to the established surgical procedure. The average (left graph) and individual (right graph in scatter plot with a linear curve fit) percent weight gain were assessed. (**P < 0.01 vs. <10 cm).

We first attempted to determine whether the surgical procedures of laparoscopically assisted myomectomy (LAM), abdominal myomectomy (AM) or total abdominal hysterectomy (TAH) affect the growth of implanted leiomyomas. Although no significant difference was found in the growth of leiomyomas at 4 weeks after implantation when LAM, AM, or TAH was performed, the implanted leiomyomas resected with AM exhibited significantly greater growth among the 3 groups at 8 weeks after implantation. (Fig. 3A) No significant difference was observed in the growth of implanted leiomyomas between those in which the central site and those in which the marginal site was sampled for tissue. (Fig. 3B) Nevertheless, since the implanted leiomyoma tissues demonstrated a trend to exhibit greater growth, we used tissues isolated from the marginal site of uterine leiomyomas in the following series of experiments. We next examined the effect of GnRHa treatment on uterine leiomyomas with respect to the growth of the implanted tissue, and revealed that GnRHa treatment (GnRHa+) significantly promoted the growth of implanted leiomyoma tissue compared with no treatment (GnRHa−). (Fig. 3C) Finally, for the growth study of implanted leiomyoma tissues, we compared the different sizes of the uterine myomas. The implanted leiomyoma tissues isolated from large (over 10 cm in diameter) myomas exhibited significantly enhanced growth compared with those from small (less than 10 cm in diameter) myomas. (Fig. 3D) The results exhibited a great deal of variation in each group because of, perhaps, randomly corrected human tissue samples with different background characteristics and variations in the conditions of individual recipient mice. Nevertheless, statistical analysis allowed us to interpret the results scientifically, and these results therefore suggest that the surgical procedure, pre-treatment of the uterine leioymoymas with GnRHa, and the original size of the uterine myomas might be critical for implanted leiomyoma tissue growth.

### GnRHa treatment was critical for the growth of implanted leiomyomas

**Figure 4:**
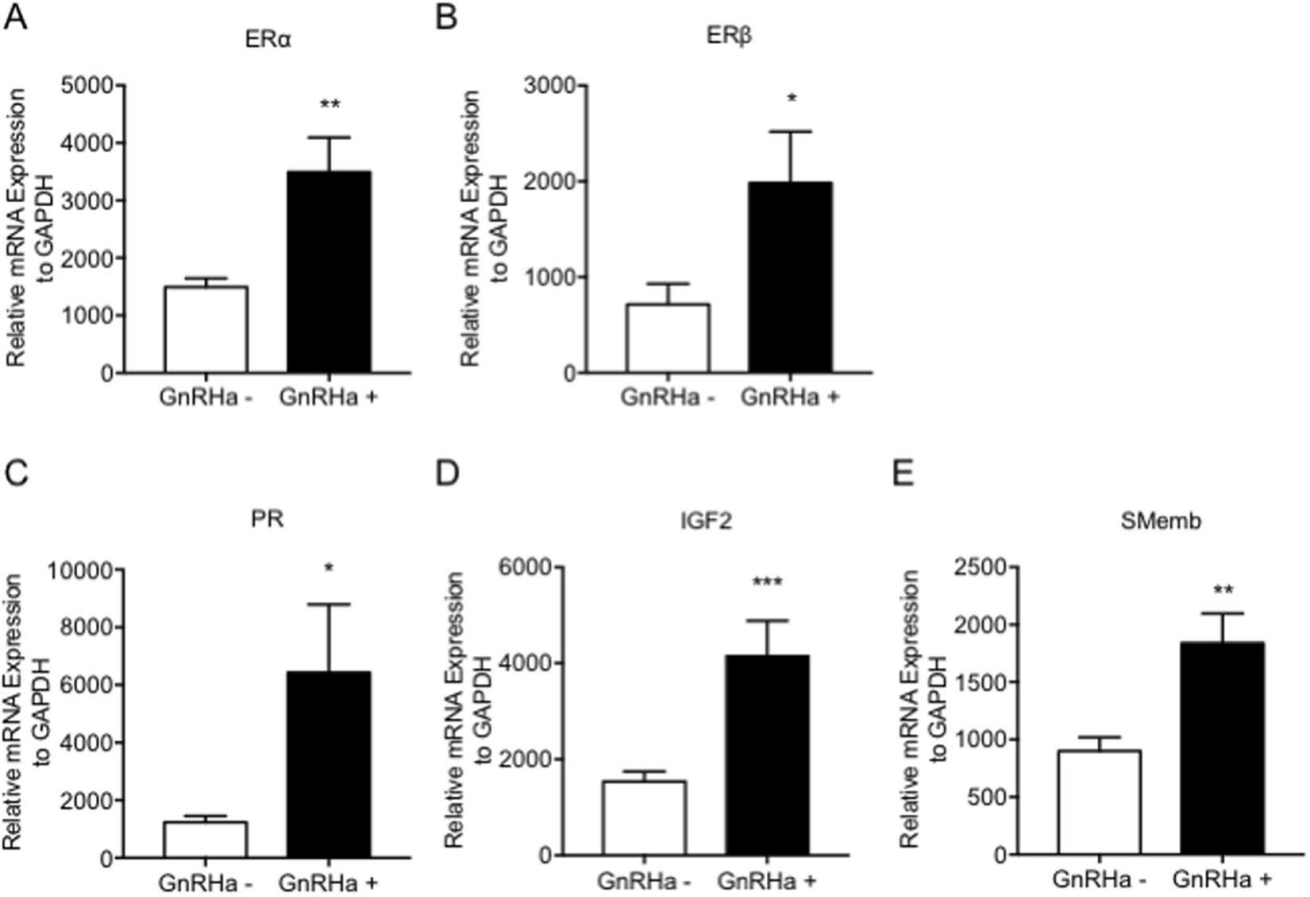
Gene expression in leiomyomas with or without GnRHa treatment. The relative mRNA expression levels of each gene were normalized to human GAPDH and were assessed by quantitative real-time RT-PCR analysis for estrogen receptor α (ERα) (**A**), estrogen receptor β (ERβ) (**B**), progesterone receptor (PR) (**C**), insulin-like growth factor 2 (IGF2) (**D**), and embryonic smooth muscle myosin heavy chain isoform (SMemb) (**E**) in leiomyomas obtained from the patients with or without GnRHa treatment. (*P < 0.05; **P < 0.01; and ***P < 0.001 vs. GnRHa−, n = 3/each group) All experiments were performed in triplicate, and the representative graph for each expressed gene is demonstrated.

**Figure 5:**
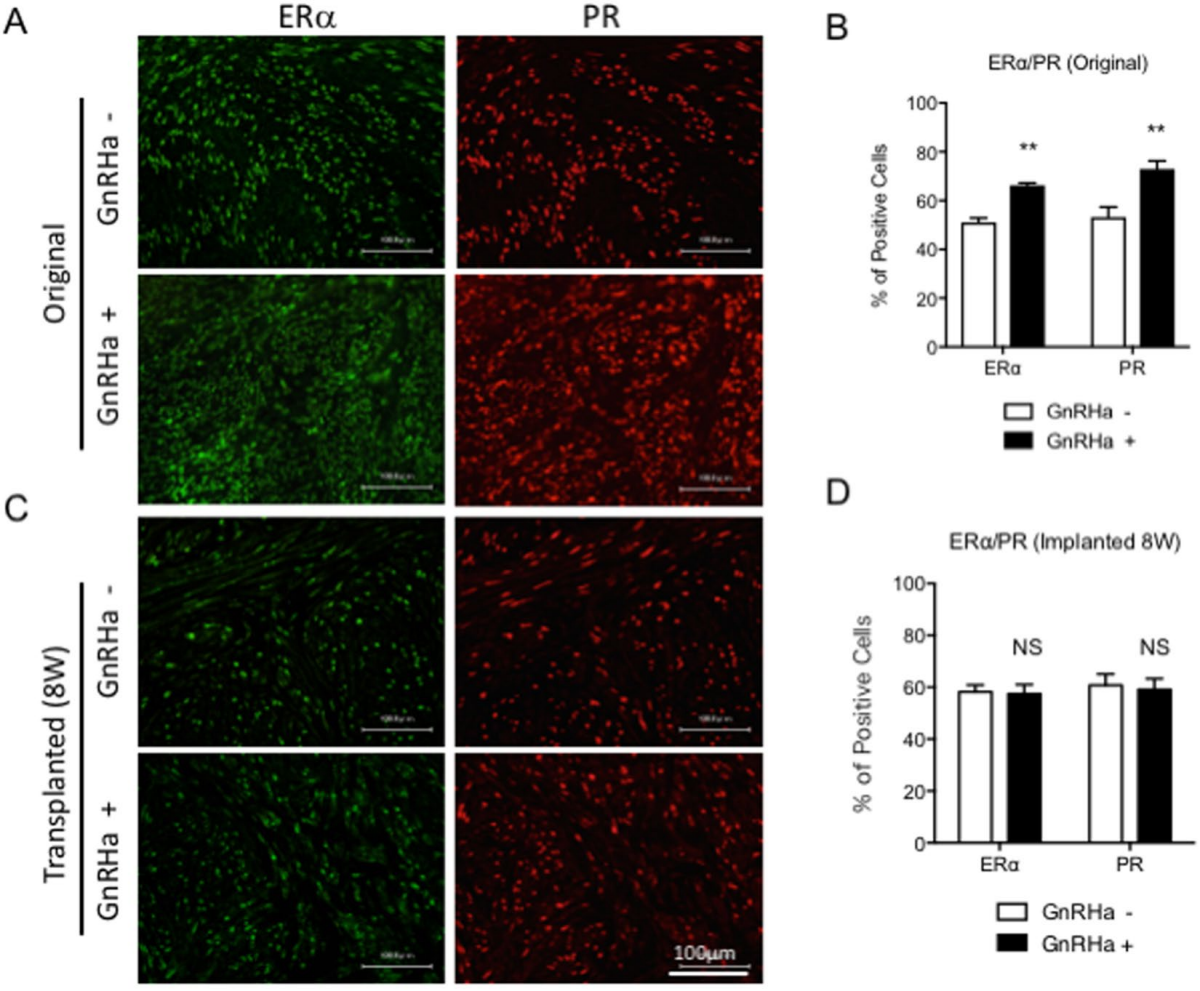
ERα and PR expression in leiomyoma with or without GnRHa treatment. Double immunofluorescence staining for ERα (A and C, left panels, shown in green) and PR (A and C, right panels, shown in red) was performed on tissue sections of leiomyoma before (Original) (A) and 8 weeks after transplantation (Transplanted (8 W)) (**C**) with or without GnRHa treatment. The quantitative analyses of ERα- and PR-positive cells in leiomyomas before (**B**), Original) and 8 weeks after transplantation (**D**), Transplanted 8 W) are demonstrated in the graphs. (NS, not significant and **P < 0.01 vs. GnRHa−, n = 3/each group) All experiments were performed in triplicate, and the representative images are presented.

We next attempted to determine how GnRHa treatment promotes the growth of implanted leiomyomas *in vivo*. The leiomyoma tissues with or without GnRHa treatment were freshly isolated from the marginal site of large myomas based on the above findings. The gene profiles were then examined by quantitative real-time RT-PCR analysis. The mRNA expression levels of estrogen receptor α (ERα), estrogen receptor β (ERβ), and progesterone receptor (PR) were significantly up-regulated in leiomyomas that were treated with GnRHa. (Fig. 4A–C) In addition, the mRNA expression of insulin-like growth factor 2 (IGF2), which is a marker of fibroids/tumour proliferation, and embryonic smooth muscle myosin heavy chain (SMemb), which is a marker of proliferating smooth muscle cells (SMCs), was significantly up-regulated in leiomyomas that were treated with GnRHa. (Fig. 4D,E) An immunohistochemical analysis of ERα and PR also showed that the number of ERα- and PR-positive cells was significantly greater in freshly isolated leiomyomas that were treated with GnRHa. (Fig. 5A,B) However, no significant differences were found in the number of ERα- and PR-positive cells in leiomyoma tissues resected 8 weeks after implantation. (Fig. 5C,D)

**Figure 6:**
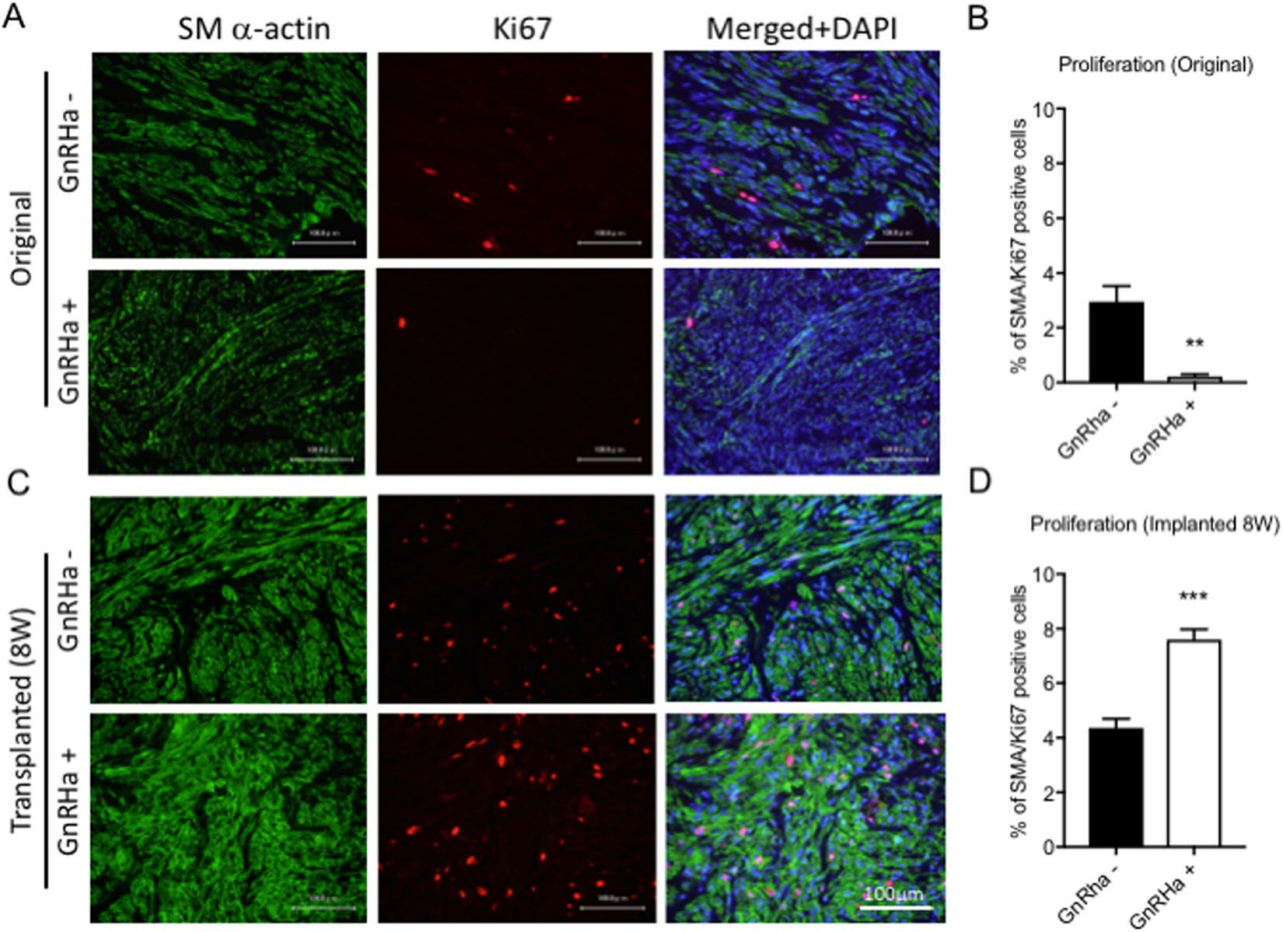
Proliferative activity in leiomyoma with or without GnRHa treatment. Double immunofluorescence staining for smooth muscle (SM) α-actin (A and C, left panels, shown in green) and Ki67 (A and C, mid panels, shown in red) was performed on tissue sections from leiomyoma before (Original) (**A**) and 8 weeks after transplantation (Transplanted (8 W)) (**C**) with or without GnRHa treatment. The merged images of SM α-actin and Ki67 with DAPI (blue) are shown in the right panels. (**A** and **C**) The quantitative analyses of SM α-actin- and Ki67-positive cells in leiomyomas before (**B**, Original) and 8 weeks after transplantation (**D**, Transplanted 8 W) are demonstrated in the graphs. (**P < 0.01 and ***P < 0.001 vs. GnRHa−, n = 3/each group) All experiments were performed in triplicate, and the representative images are presented.

To assess the proliferative activity in the implanted leiomyomas, immunostaining for Ki67 was performed in leiomyoma tissue before and 8 weeks after implantation. As expected, the number of SM α-actin/Ki67-double positive SMCs was significantly reduced in leiomyoma tissues treated with GnRHa compared with those not treated with GnRHa. (Fig. 6A,B) In contrast, the number of SM α-actin/Ki67-double positive SMCs was significantly increased in leiomyoma tissues treated with GnRHa treatment compared with those not treated with GnRHa, (Fig. 6C,D), which resulted in the development of solid uterine myomas.

## Discussion

In the present study, we established a novel mouse model of human uterine leiomyoma with the following features: (1) growing myomas, (2) a simple surgical procedure, (3) leiomyoma tissue implantation without Matrigel^TM^, and (4) an affordable and manageable mutant BALB/c-nu/nu mouse model. In the following paragraphs, we would like to discuss multiple factors that contributed to the successful development of this mouse model compared with previous similar models.

Severe immunodeficient mice (i.e., NOD-scid (NOD.CB17-*Prkdc*^*scid*^/ShiJlc) mice or NOG mice (NOD.Cg-Prkdcscid Il2rgtm1Sug/Jic))^14^ have frequently been used as recipients to avoid immunorejection and inflammation of xenografts. (Table 1) However, these mice are not resistant to stress and bacterial infection and need to be treated carefully in special housing conditions, and they are therefore not convenient for animal experiments. Thus, we decided to use BALB/c nude mice, which can be handled easily in any experiment. Matrigel^TM^ contains multiple angiogenic growth factors with matrices and has widely been used to induce angiogenesis to supply blood/nutrition for implanted tissue or cells that are engrafted in host experimental animals. However, we realized that Matrigel^TM^-induced excessive angiogenesis resulted in a reduced size of transplanted leiomyoma tissue in nude mice. The phenomenon might be explained by the finding that tumour size is reduced by tumour vascular normalization and tissue oxygenation^15^.

**Table 1:** Murine xenograft models of human uterine leiomyoma.

Transplantation		Recipient (mouse)	Growth Factor	Hormone	Growth Rate (%)	Reference
Transplant	Location					
VEGF2/COX2-overexpressing leiomyoma tissue	SC	SCID	MTG	E	130	^11^
Leiomyoma tissue	SC	NOG	MTG	E	100	^10^
Primary cultured leiomyoma cells /Leiomyoma tissue	SRC	NOG	Collagen gel/−	E + P	170/240	^23^
Primary leiomyoma cells	SC	SCID	MTG + bFGF, EGF, Insulin	E + P	N/A	^13^
Leiomyoma-derived cell line (ELT3)	SC	BALB/c nude	—	E	N/A	^24^
Leiomyoma-derived cell line (ELT3)	SC	BALB/c nude	—	—	N/A	^25^
Leiomyoma tissue	SC	BALB/c nude	—	E + P	300	Present Study

VEGF, vascular endothelial growth factor; COX2, cyclooxygenase2; bFGF, basic fibroblast growth factor; EGF, epidermal growth factor; SC, subcutaneous space; SRC, subrenal capsule; SCID, severe combined immunodeficiency; NOG, non-obese diabetic (NOD)/SCID γc-null; MTG, Matrigel^TM^; E, estradiol; P, progesterone; ELT3, Eker rat uterine leiomyoma-derived cell line.

We compared leiomyoma tissues isolated from uterine myomas that were surgically resected according to the different procedures of laparoscopically assisted myomectomy (LAM) with electric morcellator, abdominal myomectomy (AM) or total abdominal hysterectomy (TAH). The results revealed that AM might be an ideal surgical procedure for leiomyoma tissue harvest compared with LAM and TAH. The reason that the xenograft tissues obtained by LAM and TAH failed to grow after implantation might be due to mechanical tissue damage induced by the device and long-term blood occlusion of feeder arteries before hysterectomy, respectively. In regard to the size of the myoma and the sites of tissue harvesting, although no statistically significant difference was found in the capacity for growth between the central site and the marginal site in myomas, larger-sized myomas have cells in the marginal sites that are highly mitogenically active, which results in more outer growth^16,17^.

GnRHa is a standard medication given to patients with uterine myomas in order to reduce/inhibit the size/growth of tumours prior to surgical resection. Since GnRHa administration negatively regulates the production of estrogen/progesterone, which leads to a reduction in myoma size, the expression of estrogen receptor (ER) and progesterone receptor (PR) are up-regulated in leiomyoma by a negative feedback mechanism; this is consistent with what was published in previous reports^18,19^ and in the current study. Thus, the leiomyoma tissues with high expression of ER and PR can rapidly begin to grow in response to exposure to estradiol/progesterone in the hormone (E2/progesterone) pellet-implanted mice. In addition, the up-regulation of IGF2 and SMemb mRNA expression was detected in leiomyomas treated with GnRHa. IGF2 has been reported to be up-regulated by hormonal treatments with GnRHa^20^ and to be a specific tumour growth marker for leiomyomas (fibroids)^21^. For this reason, IGF2 might be associated with the development of leiomyomas. SMemb, non-muscle myosin heavy chain, is known to be a marker of immature synthetic smooth muscle cells (SMCs)^22^, and the up-regulation of SMemb leads to tumour progression via SMC proliferation in leiomyoma. Indeed, SMemb-positive SMCs or myofibroblasts were detected in marginal site of xenograft (Fig. 2H) and Ki67-positive proliferating SMCs (Fig. 6C) might also contribute to tumor growth toward outside even at 8 weeks when serum estradiol and progesterone levels are almost at baseline. These findings indicate that estrogen and progesterone might play a role as a trigger as well as required factors for SMC proliferation and may not need to be kept at high serum levels once xenograft start growing and that the SMC proliferation will last for a certain period even after the serum hormone levels return to the baseline. In terms of making a simple and easy experimental animal model, the finding that initial single hormone pellet implantation allows xenograft keep growing up to 8 weeks after implantation would be one of the advantages of this mouse model.

In conclusion, the ideal leiomyoma tissue for the development of implantable growing human leiomyoma in BALB/c nude mice should be isolated from the marginal site of large (over 10 cm in diameter) myomas treated with GnRHa and resected by abdominal myomectomy. Compared with previous models, we have developed a novel and simple mouse xenograft model of human leiomyoma despite the xenograft consists of not only human SMCs but also the cells derived from mouse (host) tissue, specifically, in central site of xenograft. (Table 1) This mouse model is an invention covered by the Japanese patent application No. 2014-030088, and would be useful for the development of therapeutic applications including the discovery of new compounds or non-invasive treatments for human uterine leiomyomas.

## Methods

### Tissue samples

Samples of uterine leiomyoma were collected from patients with multiple leiomyomas who underwent hysterectomy by laparotomy or laparoscopic surgery. This study was approved by the Osaka Medical College Ethics Committee (approved protocol No.1040-01), and all methods were performed in accordance with the relevant guidelines and regulations. Informed written consent was also obtained from all subjects. In all, 20 myoma tissue specimens were obtained from patients with uterus myomatosus who underwent laparoscopically assisted myomectomy (LAM) with electric morcellator, abdominal myomectomy (AM) or total abdominal hysterectomy (TAH). The leiomyoma tissue obtained by TAH, which was frequently performed at our institute, was used in a preliminary study to determine whether Matrigel^TM^ was necessary for implantation in mice. All patients were premenopausal with or without treatment with gonadotropin-releasing hormone agonist (GnRHa), and donors who had received hormonal or immunomodulatory therapies as well as those with known gynaecologic comorbidities, such as endometriosis, were excluded. With respect to treatment with GnRHa, leuprorelin acetate (1.88 mg/person) was administered to the patients subcutaneously 3 times at 4-week intervals before surgery. The small pieces of tissue samples were implanted subcutaneously in BALB/c nu/nu (athymic nude) mice or were analysed by histology and quantitative real-time RT-PCR.

### Estradiol/Progesterone PLGA pellet

An estradiol (E2, 1.5 mg)/Progesterone (PG, 5 mg)-poly-d, l-lactide-co-glycolide (E2/PG-PLGA) pellet with a size of φ5 mm × 2 mm/each was provided by Dr. Tabata (Kyoto University, Japan). The average molecular weight of PLGA was approximately 23,000 Da, and the monomer composition was 50 mol% lactide and 50 mol% glycolide (Wako Pure Chemical Industries Ltd., Osaka, Japan). E2 and Progesterone were purchased from Sigma-Aldrich Japan K.K. (Tokyo, Japan). Serum E2 and PG levels in mice were measured using ELISA kits (Cayman Chemical, Ann Arbor, MI, USA) according to the manufacturer’s instructions.

### Mouse xenograft model

All animal procedures were performed according to the guidelines of the Osaka Medical College Animal Care and Use Committee, and the experimental protocol was approved by the committee (protocol number 26017). Female BALB/c nu/nu mice (8–10-weeks-old, CLEA, Japan) were anesthetized with an intraperitoneal injection of 400 mg/kg 2,2,2-tribromoethanol (Avertin^TM^, Sigma-Aldrich Japan K.K., Tokyo, Japan). The small pieces of human leiomyoma tissue (4–5 mm × 4–5 mm size) were implanted with or without 50 µL of Matrigel^TM^ (BD Bioscience, Tokyo, Japan)/site in the lower dorsal skin pocket made with a skin incision 5–6 mm in length. An E2/Progesterone PLGA pellet was also implanted in the upper dorsal skin pocket made with a skin incision 6–7 mm in length (Arrow, Fig. 2A). The skin wounds were closed with three 6–0 nylon sutures and the xenograft surgery was completed. The hormone pellet was maintained until it was completely absorbed. The xenotransplanted tissue was harvested at 4 and 8 weeks after surgery. The percent of weight gain was calculated according to the formula (resected tissue weight/initial weight) × 100 at each time point, and the tissue sections were analysed by histology. The estradiol and progesterone concentrations in serum obtained from blood samples before and every week after hormone pellet implantation were measured by ELISA (Cayman Chemical, Ann Arbor, MI, USA) according to the manufacturer’s instructions.

### Quantitative real-time RT-PCR analyses

Total RNA was extracted from the placenta using an RNeasy Mini Kit (QIAGEN Science, Hilden, Germany), and reverse transcription was performed using a PrimeScript^TM^ II 1st strand cDNA Synthesis Kit (Takara Biochemicals, Kyoto, Japan) according to the manufacturer’s instructions. For quantitative RT-PCR, the converted cDNA samples (2 µL) were amplified in triplicate in a real-time PCR machine (CFX Connect, BioRad, Hercules, CA, USA) in a final volume of 10 µL using SsoFast EvaGreen Supermix reagent (BioRad, Hercules, CA, USA) with gene-specific primers. (Table 2) Melting curve analysis was performed with CFX Manager software (BioRad, Hercules, CA, USA), and the mean cycle threshold (Ct) values were used to calculate gene expression with normalization to human GAPDH.

**Table 2:** Specific primers for quantitative real-time RT-PCR amplifications.

Gene Name	Primer Sequences (5′-3′)		GenBank Accession Number
	Forward	Reverse	
ERα	CTTGCTCAGTTCTTAGTG	AATCCTCACGCTTAGTAA	NM_001122742
PR	TGATGTCTGAAGTTATTGC	CCTCATAATCCTGACCAA	NM_001271162
IGF2	CTTACCGCCCCAGTGAGACC	ACTTGGCGGGGGTAGCACAG	NM_000612
SMemb	AATACAGTGGGACAGTTA	TAAGTCTGAAGCAGGATA	S67247
GAPDH	TATGACAACAGCCTCAAG	ATGAGTCCTTCCACGATA	NM_002046

ERα: estrogen receptor α, PR: progesterone receptor, IGF2: insulin-like growth factor 2, SMemb: myosin heavy chain isoform in embryonic smooth muscle, GAPDH: glyceraldehyde-3-phosphate dehydrogenase.

### Histological assessment of uterine leiomyoma

For immunofluorescence, tissues were embedded in OCT compound (SAKURA, Tokyo, Japan) and snap-frozen in liquid nitrogen. Frozen cross sections that were 6 µm in thickness were mounted on saline-coated glass slides. After fixation in 2% PFA/PBS for 15 minutes at 4 °C, the sections were stained with primary antibodies against the following: α smooth muscle actin (Abcam, 1:200), SM22α (Abcam, 1:200), and caldesmon (Abcam, 1:50) for the assessment of smooth muscle cells, non-muscle myosin IIB (SMemb, Abcam, 1:100) for the assessment of synthetic/dedifferentiated smooth muscle cell and myofibroblast, FITC-isolectin B4 (Vector, 1:100) for the assessment of endothelial cells, estrogen receptor α (ERα, Santa Cruz Biotechnology, 1:100), progesterone receptor (PR, Dako, 1:100), Ki67 (Abcam, 1:200) for the assessment of proliferating cells, human mitochondrial ribosomal protein L11 (Abcam, 1:100) for human tissue-derived cells, and 4′,6-diamidino-2-phenylindole (DAPI: Wako Pure Chemical Industries Ltd., Osaka, Japan) to label the nuclei. Antigens were visualized with the secondary antibodies Alexa488-/Alexa594-conjugated anti mouse/rabbit IgG (BioLegend, 1:1000) and were observed under a fluorescence microscope (KEYENCE BZ-X700, Osaka, Japan). The ERα-, PR-, and Ki67-positive cells were counted in 5 selected high power fields (HPF, x100 magnification) using the ImageJ (NIH, Bethesda, MD, USA) software and were averaged for quantitative analysis.

### Statistical Analysis

All values are presented as the mean ± SEM. Statistical comparisons between 2 groups were performed by Mann-Whitney U test. Multiple groups were analysed by one-way ANOVA followed by an appropriate post hoc test (Tukey procedure) to determine statistical significance. All *in vitro* experiments were performed at least in triplicate and were then analysed. The authors had full access to the data and take full responsibility for its integrity. All authors have read and agree to the manuscript as written.
